# Development and Internal Validation of a Multivariable Prediction Model for Adrenocortical-Carcinoma-Specific Mortality

**DOI:** 10.3390/cancers12092720

**Published:** 2020-09-22

**Authors:** Madeleine H. T. Ettaieb, Sander M. J. van Kuijk, Annelies de Wit-Pastoors, Richard A. Feelders, Eleonora P. M. Corssmit, Elisabeth M. W. Eekhoff, Paul van der Valk, Henri J. L. M. Timmers, Michiel N. Kerstens, Heinz-Josef Klümpen, Rachel S. van Leeuwaarde, Bas Havekes, Harm R. Haak

**Affiliations:** 1 Department of Internal Medicine, Division of Endocrinology, Máxima Medical Center, 5631 Eindhoven/Veldhoven, The Netherlands; annelies.pastoors@gmail.com (A.d.W.-P.); H.Haak@mmc.nl (H.R.H.); 2 Department of Clinical Epidemiology and Medical Technology Assessment, Maastricht University Medical Center, 6229 Maastricht, The Netherlands; sander.van.kuijk@mumc.nl; 3 Department of Internal Medicine, Division of Endocrinology, Erasmus Medical Center, 3015 Rotterdam, The Netherlands; r.feelders@erasmusmc.nl; 4 Department of Internal Medicine, Division of Endocrinology, Leiden University Medical Center, 2333 Leiden, The Netherlands; E.P.M.van_der_Kleij-Corssmit@lumc.nl; 5 Department of Internal Medicine, Division of Endocrinology, VU Medical Center, 1081 Amsterdam, The Netherlands; emw.eekhoff@amsterdamumc.nl; 6 Department of Pathology, Location VU Medical Center, Amsterdam University Medical Centers, 1081 Amsterdam, The Netherlands; p.vandervalk@amsterdamumc.nl; 7 Department of Internal Medicine, Division of Endocrinology, Radboud University Medical Center, 6525 Nijmegen, The Netherlands; Henri.Timmers@radboudumc.nl; 8 Department of Endocrinology, University of Groningen, University Medical Center Groningen, 9713 Groningen, The Netherlands; m.n.kerstens@umcg.nl; 9 Department of Medical Oncology, Amsterdam University Medical Centers, Cancer Center Amsterdam, University of Amsterdam, 1105 Amsterdam, The Netherlands; h.klumpen@amsterdamumc.nl; 10 Department of Endocrine Oncology, University Medical Center Utrecht, 3584 Utrecht, The Netherlands; R.vanLeeuwaarde@umcutrecht.nl; 11 Department of Internal Medicine, Division of Endocrinology and Metabolism, Maastricht University Medical Center, 6229 Maastricht, The Netherlands; bas.havekes@mumc.nl; 12 Department of Internal Medicine, Division of General Internal Medicine, Maastricht University Medical Center, 6229 Maastricht, The Netherlands; 13 CAPHRI School for Public Health and Primary Care, Ageing and Long-Term Care, 6229 Maastricht, The Netherlands

**Keywords:** adrenocortical carcinoma, prediction, mortality

## Abstract

**Simple Summary:**

Adrenocortical carcinoma is a rare and aggressive cancer. Great variability in clinical course is observed, ranging from patients with extreme long survival to aggressive tumors with prompt fatal outcome. This heterogeneity in survival makes it complicated to tailor treatment strategies for an individual patient. Therefore we sought to identify prognostic factors associated with ACC specific mortality. We analyzed the data of 160 ACC patients and developed a clinical prediction model including age, modified European Network for the Study of Adrenal Tumors (mENSAT) stage, and radical resection. This easy-to-use prediction model for ACC-specific mortality has the potential to guide clinical decision making if externally validated.

**Abstract:**

Adrenocortical carcinoma (ACC) has an incidence of about 1.0 per million per year. In general, survival of patients with ACC is limited. Predicting survival outcome at time of diagnosis is a clinical challenge. The aim of this study was to develop and internally validate a clinical prediction model for ACC-specific mortality. Data for this retrospective cohort study were obtained from the nine centers of the Dutch Adrenal Network (DAN). Patients who presented with ACC between 1 January 2004 and 31 October 2013 were included. We used multivariable Cox proportional hazards regression to compute the coefficients for the prediction model. Backward stepwise elimination was performed to derive a more parsimonious model. The performance of the initial prediction model was quantified by measures of model fit, discriminative ability, and calibration. We undertook an internal validation step to counteract the possible overfitting of our model. A total of 160 patients were included in the cohort. The median survival time was 35 months, and interquartile range (IQR) 50.7 months. The multivariable modeling yielded a prediction model that included age, modified European Network for the Study of Adrenal Tumors (mENSAT) stage, and radical resection. The c-statistic was 0.77 (95% Confidence Interval: 0.72, 0.81), indicating good predictive performance. We developed a clinical prediction model for ACC-specific mortality. ACC mortality can be estimated using a relatively simple clinical prediction model with good discriminative ability and calibration.

## 1. Introduction

Adrenocortical carcinoma (ACC) is a rare malignancy with an annual incidence of about 1.0 per million individuals [1]. With an overall one-year survival rate of 60% and a five-year survival rate of 32% [1], the disease has a poor prognosis in general. Nonetheless, patients who survive for 12–28 years have been documented [2]. The exact reason for the long survival in these patients is unknown. Several factors may be responsible for these differences in survival, e.g., tumor type, lifestyle, and genetic variability. Predicting survival outcome at the time of diagnosis is difficult in clinical practice. Some clinical variables show prognostic potential: the Weiss score is the internationally acknowledged pathologic scoring system to differentiate between malignant and benign tumors. The Weiss score is a nine-point scoring system, in which a score of three or higher corresponds to a high probability of malignancy [3,4]. A Weiss score greater than six has been found to correspond to poor prognosis [5].

Another histological weighted scoring system is the Van Slooten Index (VSI). A score of eight or higher corresponds to a high probability of malignancy [6]. Unfortunately, neither of these pathological features are sufficiently specific for an estimation of prognosis in ACC.

The Ki67 index, an immunohistochemical marker, has been suggested as an additional prognostic parameter. The Ki67 index is an estimate of the percentage of tumor proliferation. A Ki67 index over 5% is suggestive of malignancy [7]. A malignant tumor with a Ki67 index under 10% is associated with a relatively good prognosis; an index over 20% is associated with an undesirable course of disease [5,8]. However, the Ki67 scoring assessment varies greatly, and both inter- and intra-observer variations cause significant limitations to its clinical utility [9]. Additionally, the clinically relevant cutoff values that are suggested to be associated with prognosis are debatable.

Other molecular or genomic prognostic markers that can clearly distinguish between low-risk and high-risk ACC tumors are being increasingly investigated [10], but their clinical value as prognostic tools has not yet been determined in large prospective series and therefore they are not part of the current clinical guidelines [11].

In 2004, the International Union Against Cancer (UICC) and the World Health Organization published the first staging classification based on the Tumor, Node, Metastasis (TNM) criteria for ACC [12]. Due to shortcomings, the European Network for the Study of Adrenal Tumors (ENSAT) developed a revised staging system [13]. The ENSAT staging system is currently recommended for estimating the prognosis of a patient with ACC [5,12]. However, the ENSAT stage is still associated with considerable heterogeneity, as reflected by a five-year stage-dependent survival of 66–82% for stage I, 58–64% for stage II, 24–50% for stage III, and 0–17% for stage IV [14]. A prediction model combining multiple parameters that have been individually associated with survival could be of significant additional value, and may be more useful in clinical practice. A clinical prediction model can inform patients and their physicians of the patients’ probability of a specified outcome and help them with associated decision making. Previous prediction models were developed for specific subgroups of ACC patients: recurrence-free (RFS) and overall survival (OS) after curative resection of ACC [15], OS of ACC patients after surgery [16], or lacking essential (histologic) predictors in the model development [17,18,19]. Therefore, the aim of this study is to develop and internally validate a multivariable, generally applicable clinical prediction model for ACC-specific mortality.

## 2. Results

**Figure 1:**
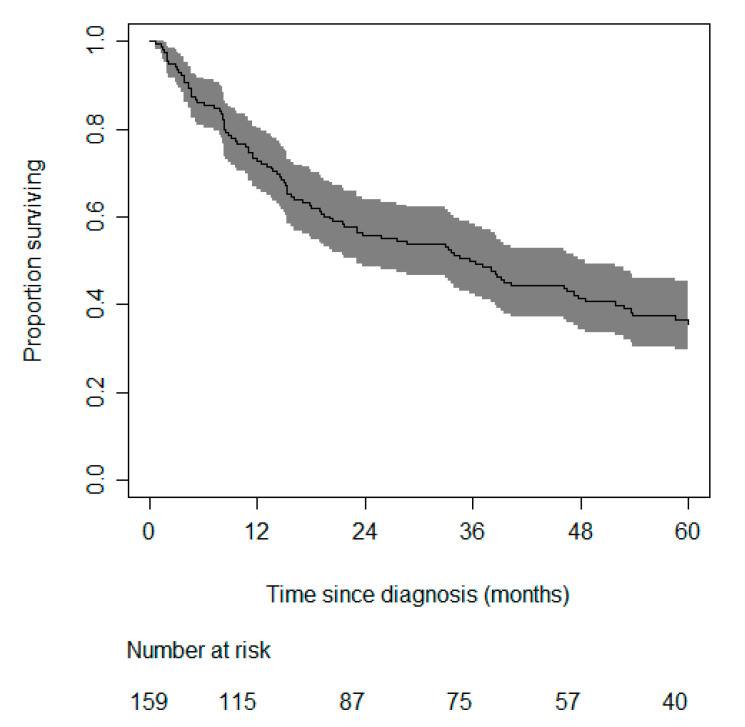
Kaplan–Meier curve, including 95% confidence band of the risk of adrenocortical carcinoma-related mortality. The numbers above the *x*-axis denote the number of patients still at risk at each point in time.

A total of 160 patients were included in the cohort. One patient was omitted from the analysis, as no outcome measures were available. Patients were followed for a median period of 33 months (1st and 3rd quartile: 11.0–61.5). A total of 108 (67.9%) patients died during the course of follow-up. The median survival time was 35.6 months (range 0.7–145.4 months). The characteristics of all patients included in this study are shown in Table 1. Figure 1 shows the Kaplan–Meier curve of the total cohort, for a total follow-up time of 60 months.

In the original database, before attempted revision, 48 (30%) out of 160 patients had a Ki67 score. Fifty-five of 160 (34%) tumor samples were retrieved for central revision of the Ki67. Of only 49 samples, the appropriate material was available for immunohistochemistry analysis and of these 49 samples 13 tumors already had a Ki67 record in the database (net: 49 − 13 = 36 new Ki67 data). Revision data were used for final analysis. The number of valid records increased from 48 (30%) in the original database to (48 + 36) 84 (52.5%) in the new database.

The correlation between ENSAT and mENSAT was high (rho = 0.93, *p* < 0.001). We selected mENSAT for further statistical modelling based on a stronger univariable association with survival. The c-statistic for mENSAT was 0.73, compared to 0.71 for ENSAT. The restricted cubic spline regression revealed no significant non-linear associations between continuous predictor variables and mortality. Because of the high proportion of missing values on the Ki67, the modelling procedure was performed both with and without Ki67.

Table 2 shows the coefficients of the predictor variables that were significantly associated with ACC-related mortality. In the model that was derived without the Ki67 index, cortisol and pathology positivity were also excluded during the backward elimination process because their *p*-values were too high (*p* = 0.469 and *p* = 0.155 upon exclusion, respectively). All predictors were risk factors for ACC-specific mortality, except for radical resection, which was protective. The scaled Schoenfeld residuals revealed that no predictor variables violated the proportional hazards assumption, indicating that the predictor variables are valid for the whole follow-up period.

Pathology was scored positive if venous invasion or capsular invasion were present according to the Weiss criteria in the pathology report or capsular and/or vascular invasion was scored yes according to the Van Slooten Index in the pathology report (see Section 4).

**Figure 2:**
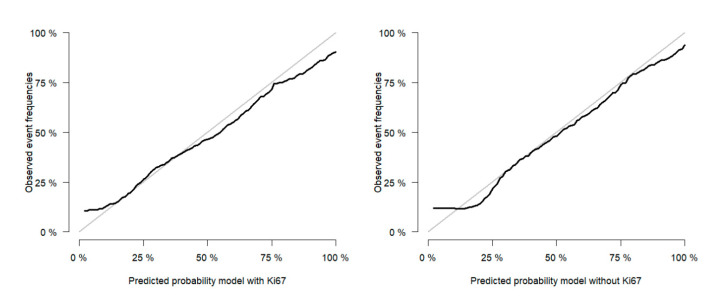
Months of follow-up time.

The c-index, which was computed to quantify the ability of the model to separate events of ACC-specific mortality from patients who did not experience the event, was 0.77 (95% CI: 0.73, 0.81) and 0.77 (95% CI: 0.72, 0.81) for the models including and excluding Ki67 in the selection process, respectively. Figure 2 shows the calibration plots of both models. The lines closely follow the ideal line of 45 degrees, indicating that both models are well-calibrated.

**Figure 3:**
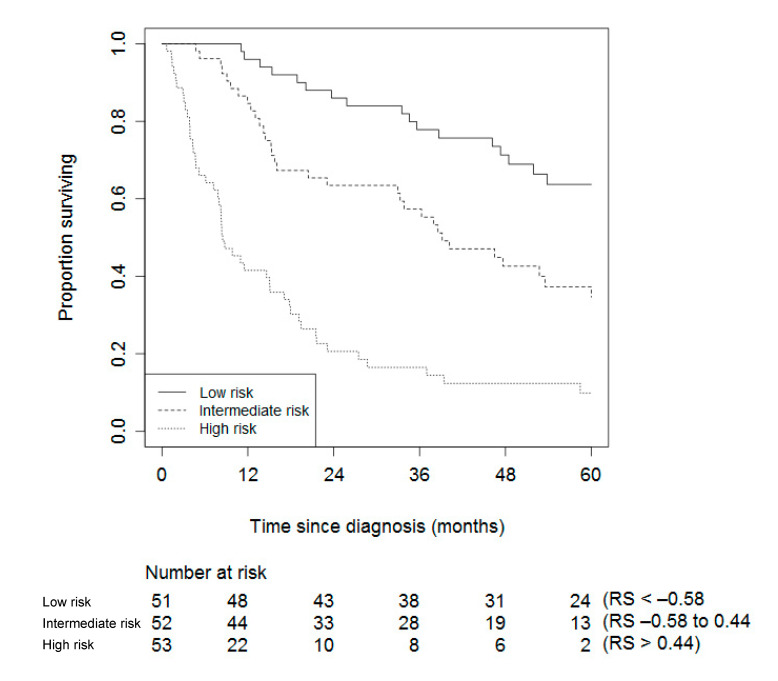
Kaplan–Meier stratified by risk groups based on each individual’s risk score (RS) of the model including Ki67.

**Figure 4:**
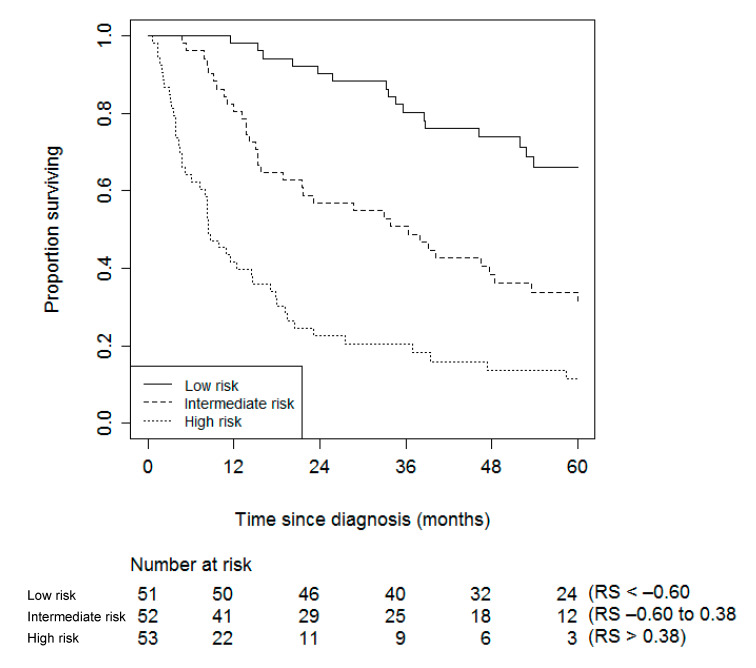
Kaplan–Meier stratified by risk groups based on each individual’s risk score (RS) of the model without Ki67. The Risk Score can be computed as 0.02 × (age − 54.5) + 0.63 × (mENSAT − 3.2) − 0.44 × (radical resection of the tumor − 0.8).

The internal validation yielded a shrinkage factor of 0.91 for the model with Ki67, and 0.95 for the model without Ki67 (Table 2). Hence, slightly more overfitting was present in the model that included Ki67. In addition, the internal validation yielded a measure of optimism of the c-index of only 0.01, indicating that the c-index as a measure of the model’s ability to discriminate in future patients is estimated to be 0.76 and 0.76 for the two models, respectively (compared to the apparent ability to discriminate of 0.77 and 0.77, respectively). The shrinkage factors that are close to 1, together with the small measures of optimism, indicate that these models are not much overfitted. Figure 3 and Figure 4 show the Kaplan–Meier curves for patients stratified by their risk score, for both models.

### Prediction for Future Patients

The prediction models presented in Table 2 can either be used to compute an individual risk score to determine risk category (Figure 3 and Figure 4), or to compute an actual probability that an individual experiences ACC-specific mortality within one, two, or five years. How to do so is outlined below.

The risk score (RS) can be computed by multiplying the shrunk coefficients by the values of an individual patient minus the sample average for age (54.5), mENSAT (3.2), and radical resection (0.8). mENSAT takes on a score from 1–6, with mENSAT I-III scoring 1–3 points, and IVa-IVc scoring 4–6 points. The surgical resection is scored one if radical and zero if not (see Section 4), for example, using the model without Ki67: 0.02 × (age − 54.5) + 0.63 × (mENSAT − 3.2) − 0.44 × (radical resection − 0.8) of the tumor (no = 0, yes = 1). A 64-year old patient with an mENSAT stage of 4a, and no radical resection of the tumor, would have a risk score of 0.02 × (64 − 54.5) + 0.63 × (4 − 3.2) − 0.44 × (0 − 0.8) = 1.046. Figure 4 shows that this individual would be classified as being at high risk of ACC-specific mortality.

The probability of ACC-specific mortality within one, two, or five years is computed by combining the Kaplan–Meier estimate at one, two, or five years and the sum of the shrunk coefficients to the centered values of an individual patient (i.e., the value minus the sample average) as 1 − S(t)^exp (LP)^: S(t) is the survival function in which t is time. S(t) for one, two, and five years is 0.73, 0.56, and 0.37, respectively (see Kaplan–Meier Figure 1). Exp(LP) stands for *e* raised to the power of the linear predictor (exp: exponential (function), *e* is the mathematical constant, lp: lineair predictor). The linear predictor can be computed as 0.02 × (age − 54.5) + 0.63 × (mENSAT − 3.2) − 0.44 × (radical resection of the tumor − 0.8). The 64-year old patient with an mENSAT stage of 4a, and no radical resection (no = 0) of the tumor, would have a two-year probability of ACC-specific mortality of 1 − 0.56^exp(LP)^. LP = 0.02 × (64 − 54.5) + 0.63 × (4 − 3.2) − 0.44 × (0 − 0.8) = 1.046; hence, the probability = 1 − 0.56^exp(1.046)^ = 0.81 = 81%. This can be confirmed in Figure 4, in which approximately 20% of the high-risk group survives past 24 months.

## 3. Discussion

In this collaborative study of the Dutch Adrenal Network, a model capable of predicting ACC-specific mortality was developed. An accurate prediction model could help to identify patients at greater risk of death, and support the decision making on early systemic therapy. Furthermore, a prediction model could support selection of a specific subgroup eligible for new therapeutic compounds.

Ki67 is a suggested prognostic marker in ACC [5,20]; therefore, we performed modelling both with and without Ki67. Both models were based on tumor stage defined by the mENSAT classification, age, and radical resection of the primary tumor. The model with Ki67 also relied on hormonal status and the pathology criteria capsular and/or vascular invasion. Although both models showed comparable discriminative ability and calibration, the model with Ki67 was partially based on imputed data because of missing data despite our efforts to revise the Ki67 index for all patients. Previous research has shown that pathology data are often incompletely described in ACC [21]. In addition, pathology reports are not standardized in ACC. Prognostic scoring systems based on the Weiss and Van Slooten criteria as well as the Ki-67 are to a certain extent subjective, as a reliable assessment largely depends on the expertise of the pathologist [22]. Furthermore, there is often significant inter-observer variability in the determination of the Ki67 [9]. Therefore, in order to improve the generalizability of our prediction model, we decided to use more reliable and easily accessible clinical variables such as age, mENSAT, and completeness of the resection.

This is the first time the mENSAT stage has been considered in a prediction model. Both ENSAT stage, number of affected organs, presence of metastasis, as well as nodal status have shown to be correlated with survival [5,13,23,24,25]. The mENSAT stage combines those variables in one staging system, and considers the number of affected organs, including the primary tumor and lymph nodes [5]. The important difference compared with the ENSAT staging system currently used is the fact that T3-4N1M0 is considered stage IV instead of stage III. Lymph node positive disease has been demonstrated to be associated with a less favorable prognosis [26,27], and consequently, given the high recurrence rates for ENSAT stage III, a more prominent role for neo- and adjuvant therapy has been put forward [27]. We endorse using the mENSAT stage in clinical practice.

Estimating survival with the Kaplan–Meier analysis, which is commonly done, has its limitations. It is a nonparametric approach to survival outcomes, and it is able to show univariate relationships graphically or to compute survival factions at a certain time of follow-up. However, the Kaplan–Meier method and the log-rank test cannot be used for multivariate analysis. When looking at current data based on Kaplan Meier data, a stage IV patient could have zero percent change of 5-year survival or almost 20%. Most patients want to know if they have this small chance at 5-year survival or no chance at all when deciding on starting chemotherapy or mitotane. A clinical prediction model provides such tailored estimation on prognosis. In daily practice, a physician would like to estimate the prognosis tailored for a particular patient, underscoring the need for a reliable clinical prediction model.

In contrast to previously proposed prediction models for ACC, we included pathological data, imputed missing data to prevent a loss of statistical precision, and considered the most recently proposed mENSAT staging system [5]. Another strength of the present study is that our modelling was not based on a pre-selected group of patients with ACC, as our sample included all ENSAT stages. Kim et al. [24] developed a prediction model based on a multi-institutional group of patients treated in the United States who underwent surgery for ACC (Table 3). Notably, they excluded patients with metastatic disease at presentation as well as patients with a macroscopically nonradical resection (R2). Although they studied a relatively large cohort (*n* = 148), the external validity of their study is limited because up to 53% of patients with ACC may present with metastatic disease [21]. Even if their model was solely meant to predict RFS and OS after surgery, they still excluded patients. Surgery is being considered in patients with metastatic disease, and it has been shown that surgery may even improve the outcome in selected patients with stage IV disease, especially if an R0 resection can be achieved [28].

Li et al. presented a nomogram for overall survival (age, year of diagnosis); histologic grade (I + II, III + IV, and unknown); historic stage (localized, regional, distant, and unknown); chemotherapy (no/unknown or yes); and cancer-specific survival (CSS), including age, year of diagnosis, historic stage and chemotherapy [17]. Kong et al. did not include any histological criteria, but their nomogram included age and TNM stage (according to the 7th American Joint Committee on Cancer (AJCC) TNM staging) [16]. With the nomogram of Li et al., future use causes a problem where year of diagnosis can only be scored till 2015. In addition, their other predictors in that nomogram are not commonly used in clinical practice [17]. The same limitation applies to the nomogram by Kong et al., who use the AJCC TNM staging [16]. Age, however, is a predictor in both models.

It is interesting to note that although the work of Zini et al. [19] suffered from lack of detailed prognostic information, their prediction model included age, stage, and surgery status. In our analysis, we actually include a broad selection of potential predictor variables, and confirm age, stage, and completeness of tumor resection to be significant predictors for ACC specific mortality. So, even in the absence of detailed information, e.g., patients of whom no immunohistochemistry is available, it is possible to make an estimate of cancer-specific mortality.

The study by Kebebew et al. [29] was not designed to develop a prediction model, but their highly powered (*n* = 725) multivariable analysis for ACC mortality also showed that ACC stage, surgical resection, and tumor grade (localized, regional, or distant) were independent prognostic factors.

There is a lack of consensus regarding the cut-off point and combination of pathologic criteria that are associated with prognosis [20,30,31]. Consequently, there are limitations to the clinical use of pathology criteria for ACC prognosis. Perhaps in the near future, results of genome and transcriptome studies could be useful. The results of their potential prognostic value seem promising [10,32,33,34]. For example, hypermethylation of CpG islands, or as a pattern called CpG island methylator phenotype (CIMP), is associated with a poor prognosis in ACC [10,33,35]. At the moment, a clearly defined picture of CIMP in ACC is lacking, and although study results on CIMP patterns demonstrate a certain degree of overlap, there are some (methodological) inconsistencies. Furthermore, it is uncertain whether methylation status has the potential to become a prognostic factor on its own, or if it will be an additive to a clinical prediction model, as presented in this study. The latter might be expected considering the fact that DNA methylation is a dynamic process with potential fluctuations over time, and with differences between primary tumor and metastasis. Libbert et al. recently presented a COMBI score, integrating clinical predictors with number of somatic mutations, alterations in the Wnt/beta-catenin and p53/Rb pathways, and promoter region methylation pattern. Again, heterogeneity of molecular analysis and definition of cut-off values used pose a problem, but it could be useful as a prognostic determinant in the future.

Individual biomarkers like *G0S2* [34] and *BUB1*, *PINK1* [36,37] might overcome the problem of heterogeneity with CIMP, but those biomarkers merely identify ACC with a poor prognosis.

Some limitations of our study merit further mentioning. The prediction model is only applicable to ACC patients of 18 years or older. The cohort we used had a relatively small sample size, which limited the number of predictors for consideration in our model. In view of the fact that the Netherlands currently holds 17 million inhabitants, the size of our study cohort is in agreement with the reported incidence of ACC. In our opinion, the prediction model presented here has the potential to provide a more individualized and practical estimate of the prognosis of ACC, compared to the currently used staging system. Although our model was internally validated using bootstrap validation, external validation is warranted before widespread implementation of the algorithm in a user-friendly prediction calculator.

## 4. Materials and Methods

Data for this retrospective cohort study were obtained from the nine centers of the Dutch Adrenal Network (DAN), as has been described earlier [23]. Patients of ≥18 years who presented with ACC between 1 January 2004 and 31 October 2013 were included. Follow-up data was investigated until 30 June 2016. Because of missing Ki67 data in the original database, an attempt was made to revise the Ki67 index, from 31 January 2018 until 2 April 2020.

### 4.1. Potential Predictor Variables

We identified potential predictor variables based on clinical reasoning, and on previously published risk factors for mortality. These variables were age at the time of diagnosis [38], sex, body mass index (BMI), TNM classification, hypercortisolism, complaints directly related to tumor mass, venous invasion, ENSAT stage, modified ENSAT (mENSAT) stage [5], radical resection of the tumor (R0 variable ‘’yes’’; Rx/R1/R2 variable ‘’no’’) [20,26], and Ki67 index.

ENSAT stage was defined according to Fassnacht et al. [13]: stage I, tumor size ≤5 cm (T1N0M0); stage II, tumor size >5 cm (T2N0M0); stage III, tumor of any size with at least one of the following factors: tumor infiltration in surrounding tissue (T3), tumor invasion into adjacent organs, or venous tumor thrombus in the vena cava or renal vein (T4), positive lymph node (N1), but no distant metastasis (M0); and stage IV, the presence of distant metastases irrespective of tumor size or lymph node status (T1-T4N0-N1M1). The mENSAT classification defines T3-4N0M0 as stage III (invasion of surrounding tissues/organs, or invasion of the renal vein or inferior vena cava), and both T3-4N1M0-1 and T3-4N0M1 as stage IV. Stage IV is then subcategorized into Stage IVa (two involved organs), IVb (three involved organs), and IVc (>three involved organs). The primary tumor and ‘’N’’ are included as ‘’organ’’ in the count of number of involved organs [5].

Pathology was scored positive if venous invasion or capsular invasion were present according to the Weiss criteria in the pathology report or capsular and/or vascular invasion was scored yes according to the Van Slooten Index in the pathology report.

Hypercortisolism was defined clinically if this was reported by the treating physician in the patients’ file or biochemical with a cortisol level above the upper limit of normal as defined by the hospital laboratory where the patients’ cortisol level was analyzed. Cortisol level was determined either in serum, saliva, 24-h urine, and/or during a dexamethasone suppression test.

Complaints due to tumor mass were scored yes if the patient had abdominal pain or back pain that could be due/directly related to the tumor mass.

### 4.2. Immunohistochemistry Revision Ki67

An attempt was made to revise the Ki67 index for all 160 patients. We were able to track down 55 tumor samples.

To assess the number of cells in cycle (preparing for cell division), selected blocks were stained at one location using the MIB-1 antibody (obtained from Dako, Glostrup, Denmark). All sections were stained in an immunostainer (Ventana Benchmark Ultra, from Roche, Tucson, AZ, US) in 3 runs. Sections were developed using the Optiview 3,3-diaminobenzidine (DAB) system (also from Roche) as a second step and DAB as chromogen. To assess the number of stained cells, the area with the highest staining intensity was searched for, and that area was magnified using a 40× objective (hot spot method). All cells and all positive cells were counted, and the percentage of positive cells was calculated. A cell was deemed positive when the nucleus was no longer blue. Additionally, faint staining was included as positivity. As a control for the staining effectivity, a tissue microarray was mounted on each individual stained slide.

Unfortunately, information on the Ki67 analytic process used for the other tumor samples in the database is not available because of historical data.

### 4.3. Model Development

We chose to impute our data to prevent a loss of statistical precision and to decrease the likelihood of obtaining biased results [39]. Imputation was performed using stochastic regression imputation with fully conditional specification. Predictive mean matching was used to draw the values to be imputed.

Baseline characteristics of the participants were described using means and standard deviations or absolute numbers and percentages. The overall survival of our cohort was estimated using the Kaplan–Meier method. Median follow-up time was computed, including the first and third quartiles. The median survival time was estimated including 95% confidence interval (CI) around the median.

We computed a correlation matrix to assess correlations between predictor variables. If variables were highly correlated (Pearson’s correlation coefficient, or rho, >0.8), we chose to include only the variable that was deemed more convenient to implement in a clinical prediction tool.

Multivariable Cox proportional hazards regression was used to estimate the coefficients in the prediction model. Backward stepwise elimination was performed to derive a more parsimonious model. We used the Akaike Information Criterion as the rule for deleting variables from the model, which corresponds to a more liberal alpha of 0.157 [40]. The correlation between the scaled Schoenfeld residuals and time was computed for each variable to test the proportional hazards assumption [41].

The performance of the initial prediction model was quantified by measures of discriminative ability and calibration. Discriminative ability is expressed as the c-index, which can take on any value between 0.5 (no discriminative ability) and 1 (perfect discriminative ability), and is an estimate of the probability that of any two randomly chosen patients, the one with the higher prognostic score will outlive the one with the lower prognostic score [41]. Calibration was assessed by visual inspection of the calibration plot. The calibration plot shows the agreement between predicted probabilities and pseudo-observed event status at a follow-up time of two years.

### 4.4. Internal Validation of the Model

As a rule of thumb, it is suggested that for each potential predictor variable in the model, 10 events should be observed to prevent overfitting. An overfitted model would perform well in the development data, but poorly when applied to new patients. Often, such an overfitted model would produce too extreme predictions. Since our dataset was relatively small, an internal validation step was performed to counteract the possible overfitting of our model to the data. We used standard bootstrapping techniques (B = 1000) to obtain optimism-corrected measures of performance (the c-index) and a shrinkage factor. The shrinkage factor is calculated as the optimism-corrected calibration slope. It is a constant between 0 and 1, and the regression coefficients are then shrunk by multiplying by this number. These penalized regression coefficients produce fewer extreme predictions, and hence counteract the effect of overfitting.

Finally, we stratified all patients into three groups based on their predicted risk score (i.e., low, medium, and high risk) for both the model with and the model without the Ki67 index.

## 5. Conclusions

In conclusion, we have developed and internally validated an easy-to-use prediction model for ACC-specific mortality; this model is essentially based on age, mENSAT stage, and completeness of tumor resection.

## Figures and Tables

**Table 1:** Patient characteristics (*N* = 160) *.

	Original Data	Imputed Data
	*N* (% of total) (Range)	*N* (% of total) (Range)
Male	61 (38)	61 (38)
Female	99 (62)	99 (62)
Age at diagnosis	55 (19–89)	55 (19–89)
ENSAT Stage		
I	9 (6)	9 (6)
II	58 (36)	58 (36)
III	42 (26)	42 (26)
IV	51 (32)	51 (32)
mENSAT Stage		
I	9 (6)	9 (6)
II	58 (36)	58 (36)
III	30 (19)	30 (19)
IVa	36 (22)	36 (22)
IVb	17 (11)	17 (11)
IVc	10 (6)	10 (6)
Resection status		
R0	74 (46,25)	74 (46)
R1/R2/Rx	61 (38,1)	68 (43)
No surgery	18 (11,25)	18 (11)
Missing	7 (4,4)	-
Ki67		
≤5	27 (17)	47 (29)
6–10	19 (12)	28 (18)
11–15	8 (5)	16 (10)
>15	30 (18,5)	69 (43)
Missing	76 (47,5)	-
Capsular and/or vascular invasion		
Yes	90 (56)	125 (78)
No	24 (15)	35 (22)
Missing	46 (29)	-
Hypercortisolism		
Yes	88 (55)	88 (55)
No	35 (22)	72 (45)
Missing	37 (23)	-
Complaints due to tumor mass		
Yes	78	78
No	13	82
Missing	69	-

* To prevent a loss of statistical precision and to decrease the likelihood of obtaining biased results, we imputed the available data (see methods for further explanation). See Section 4 for definition of the predictor variables.

**Table 2:** Initial and internally validated coefficients of the prediction model for ACC-specific mortality.

Predictor	Model with Ki67			Model without Ki67		
	Coefficient	HR (95% CI)	Shrunk Coefficient *	Coefficient	HR (95% CI)	Shrunk Coefficient *
Age at diagnosis (year)	0.02	1.02 (1.01, 1.03)	0.02	0.02	1.02 (1.00, 1.03)	0.02
Pathology positive (yes)	0.52	1.68 (0.98, 2.89)	0.47			
mENSAT (stage)	0.61	1.84 (1.55, 2.18)	0.65	0.66	1.93 (1.64, 2.28)	0.63
Ki67 (%)	0.01	1.01 (1.00, 1.03)	0.01			
Radical resection of tumor (yes)	−0.37	0.69 (0.46, 1.04)	−0.34	−0.46	0.63 (0.42, 0.94)	−0.44

* Internally validated (*shrunk*) coefficients were obtained by multiplying the coefficients by the shrinkage factor of 0.91 for the model with Ki67, and 0.95 for the model without Ki67.

**Table 3:** Overview of current models for prognostication in patient with ACC.

Study	*N*			Outcome	Predictors	C Statistics
	Model development	Internal validation	External validation			
Zini et al., 2009 [19]	205	-	207	CSM and ACM in patients managed with either surgery or no surgery for ACC.	CSM/ACM: age, stage (localized, regional, and distant), and surgical status (surgery or no surgery).	-
Kim et al., 2016 [24]	148	Bootstrap validation with 200 resamplings.	-	RFS and OS after curative surgical resection of ACC.	RFS: tumor size (<12 or ≥12 cm), nodal status (N0, N1, or Nx), T stage (I/II or III/IV), cortisol-secreting tumor, and capsular invasion.	RFS: 0.74
					OS: tumor size (<12 or ≥12 cm), nodal status (N0, N1, or Nx), and resection margin (R0 or R1).	OS: 0.70
Li et al., 2018 [17]	751	Bootstrap validation with 200 resamplings.	-	OS and CSS in patients with ACC.	OS: age at diagnosis, year of diagnosis (1973–1987, 1988–2001, 2002–2015), histologic grade (I + II, III + IV, unknown), historic stage (localized, regional, distant, unknown), and chemotherapy (no/unknown, yes).	OS: 0.677
					CSS: age at diagnosis, year of diagnosis (1973–1987, 1988–2001, 2002–2015), historic stage (localized, regional, distant, and unknown), and chemotherapy (no/unknown, yes).	CSS: 0.672
Kong et al., 2019 [16]	404	318, and bootstrap validation with 1000 resamplings.	82 + 82 * = 164	OS in patients with ACC after surgery.	OS: age, T stage (T1-T4), N stage (N0, N1), M stage (M0, M1).	OS: 0.715

ACC: adrenocortical carcinoma; ACM: all-cause mortality; CSM: cancer-specific mortality; CSS: cancer-specific survival; OS: overall survival; RFS: recurrence-free survival. T, tumor. N, lymph node. M, metastasis. N0, no positive lymph nodes; N1, positive lymph node(s); Nx, not harvested. M0, no distant metastases; M1, presence of distant metastasis. Complete resection (R0); microscopically irradical (R1).* two external validation sets were used: the Cancer Genome Atlas set and a Chinese multicenter cohort dataset.
